# Controllable positive exchange bias via redox-driven oxygen migration

**DOI:** 10.1038/ncomms11050

**Published:** 2016-03-21

**Authors:** Dustin A. Gilbert, Justin Olamit, Randy K. Dumas, B. J. Kirby, Alexander J. Grutter, Brian B. Maranville, Elke Arenholz, Julie A. Borchers, Kai Liu

**Affiliations:** 1 Physics Department, University of California, Davis, One Shields Avenue, Davis, California 95616, USA; 2 NIST Center for Neutron Research, Gaithersburg, Maryland 20899, USA; 3 Department of Physics, University of Gothenburg, Gothenburg 412 96, Sweden; 4 Advanced Light Source, Lawrence Berkeley National Laboratory, Berkeley, California 94720, USA

## Abstract

Ionic transport in metal/oxide heterostructures offers a highly effective means to tailor material properties via modification of the interfacial characteristics. However, direct observation of ionic motion under buried interfaces and demonstration of its correlation with physical properties has been challenging. Using the strong oxygen affinity of gadolinium, we design a model system of Gd_*x*_Fe_1−*x*_/NiCoO bilayer films, where the oxygen migration is observed and manifested in a controlled positive exchange bias over a relatively small cooling field range. The exchange bias characteristics are shown to be the result of an interfacial layer of elemental nickel and cobalt, a few nanometres in thickness, whose moments are larger than expected from uncompensated NiCoO moments. This interface layer is attributed to a redox-driven oxygen migration from NiCoO to the gadolinium, during growth or soon after. These results demonstrate an effective path to tailoring the interfacial characteristics and interlayer exchange coupling in metal/oxide heterostructures.

## Abstract (web summary)

Direct observation of ionic motion in buried metal/oxide interfaces and its correlation with physical properties is a challenging task. Here, the authors observe oxygen migration in a model system with controllable positive exchange bias, due to the redox-driven formation of a ferromagnetic interfacial layer.

## Graphical abstract

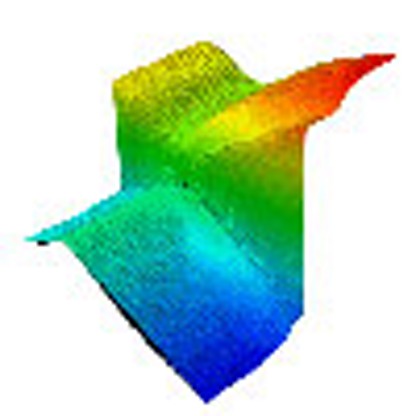

Modification of metal/oxide heterostructures through ionic motion is highly effective in tailoring the interfacial characteristics and consequently their physical and chemical properties^1^. For example, forced oxygen migration has been explored in resistive switching of memristors^2^,^3^ and control over metal–insulator transitions in electrolyte-gated materials^4^; charge-trapping has been demonstrated to substantially enhance the efficiency of magnetoelectric effects^5^,^6^. Most recently, the role of oxygen migration-induced surface chemistry modification has been highlighted in electrical tuning of interfacial magnetic anisotropy^7^,^8^,^9^,^10^,^11^, which are highly relevant to energy-efficient magnetization switching in magnetic tunnel junctions and other spintronic devices^12^,^13^. In particular, gadolinium oxide films have been used as a source of ionic oxygen, which can then be driven into a neighbouring ferromagnet (FM) with an electric field^6^,^10^,^11^. The strong oxygen affinity and preferred oxidation state of gadolinium (almost exclusively +3) plays a key role in mobilizing any off-stoichiometry oxygen. This offers an opportunity to design a system with an inverted construction, whereby, relying on the gadolinium oxygen affinity, a neighbouring oxide film is reduced. To date, however, direct observations of the oxygen migration under buried interfaces and its correlation with the physical properties in metal/oxide heterostructures have been few and far between^10^,^11^,^14^.

In this work, we demonstrate effective magneto-ionic manipulation of metal/oxide interfaces using an inverted gadolinium-based heterostructure design, manifested through the interface-sensitive exchange bias effect. We report direct evidence of controllable positive exchange bias in bilayer films of GdFe/NiCoO (ferrimagnet/antiferromagnet (AF)) enabled by the redox-driven oxygen migration. The exchange bias phenomenon is central to spin-valve type of spintronic devices^15^,^16^,^17^ and to several emerging frontiers such as multiferroics^18^,^19^, chiral ordering and spin texture^20^,^21^, control of quantum magnets^22^ and AF spintronics^23^,^24^. Conventionally, positive exchange bias, most notably in TM/TMF_2_ (TM(F)=FM transition metal (fluoride)), is due to the competition between the Zeeman energy and an AF interfacial exchange coupling, and often requires a large cooling field (on the order of a few Tesla) to realize^25^. The present GdFe/NiCoO films exhibit a full range of controllability, both the sign and magnitude of the exchange bias, using a much smaller cooling field range that is an order of magnitude smaller than previous observations^25^, along with a variable GdFe composition that affects its Curie temperature. Depth profiling with polarized neutron reflectometry (PNR) directly identifies an FM interfacial layer, a few nanometres in thickness, whose moments are substantially larger than typically expected from uncompensated AF interfacial moment alone. Using element-specific X-ray magnetic circular dichroism (XMCD) spectroscopy, the interfacial layer is shown to result from rotatable NiCo and have exchange coupling both to the adjacent GdFe and NiCoO. Thermodynamic considerations suggest that a Gd-NiCoO redox reaction causes the formation of the interfacial NiCo, which is manifested in the controllable positive exchange bias. These results provide important insights into the emerging field of magneto-ionics.

## Results

### Magnetometry

**Figure 1:**
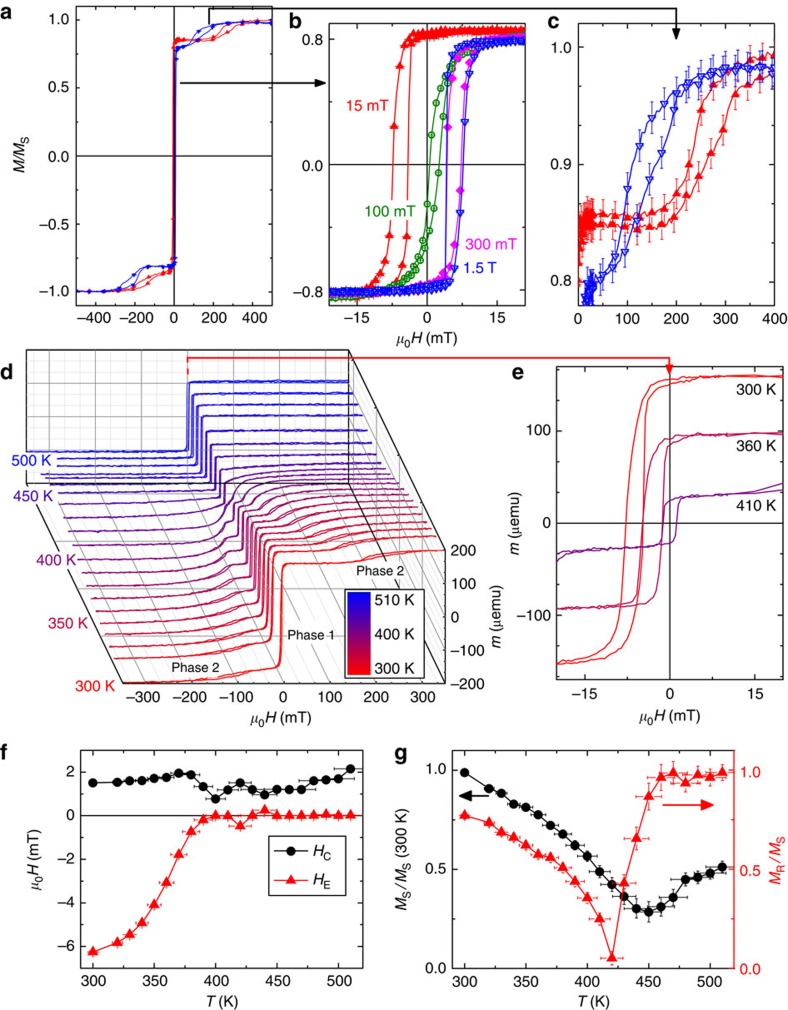
Magnetometry results for sample A (Gd_0.42_Fe_0.58_/NiCoO). (**a**) Room-temperature hysteresis loops under different cooling fields. (**b**,**c**) Zoomed-in views of phase 1 and phase 2, respectively, as indicated by arrows. The colored loops in **a**–**c** correspond to cooling fields of 15 mT (red), 100 mT (green), 300 mT (pink), and 1.5 T (blue). Temperature-dependent (**d**) hysteresis loop (300–510 K, marked by the scale bar, with a zoomed-in view of the phase 1 loops shown in **e**), (**f**) coercivity and bias, and (**g**) saturation magnetization and squareness are shown after field cooling in 15 mT. Error bars are determined by the machine sensitivity limits. For clarity, not all data points are shown.

Magnetometry measurements were performed on thin films of Ni_0.47_Co_0.53_O (20 nm)/Gd_*x*_Fe_1−*x*_ (30 nm), where *x*=0.42, 0.48, 0.53 and 0.57, identified as samples A–D, respectively (see Methods). Henceforth, for simplicity, Ni_0.47_Co_0.53_O and Gd_*x*_Fe_1−*x*_ will be identified as NiCoO and GdFe, respectively. The GdFe Curie temperature *T*_C_ was measured to be around 480 K for samples A and B, and around 350 K for samples C and D. To establish exchange bias, the samples were heated to 420 K (above the AF NiCoO Néel temperature of *T*_N_=401 K)^26^,^27^ in a helium flow furnace and then cooled to room temperature in the presence of an in-plane cooling field *μ*_o_*H*_FC_. Room-temperature hysteresis loops for sample A (*x*=0.42) are shown in Fig. 1a, after field cooling in 15, 100 and 300 mT, and 1.5 T. A small vertical shift of 1–2% of the saturation magnetization *M*_S_ is observed in the loops, resulting from uncompensated pinned moments at the AF interface^28^,^29^. Two magnetic phases are evident: a single loop at low fields with a small coercivity and a pair of asymmetrically biased subloops at higher fields (zoomed-in views shown in Fig. 1b,c, referred to as phase 1 and 2, respectively). When the sample is field cooled in small *μ*_o_*H*_FC_ (< 100 mT), the phase 1 subloop is biased in the *−H* direction and the phase 2 subloops are asymmetrically biased to the *+H* direction. As *H*_FC_ is increased, the phase 1 subloop gradually shifts from negative to positive bias, while the two subloops of phase 2 collectively shift to the −*H* direction. These trends illustrate controllable, yet opposite, exchange biases experienced by the two phases under increasing cooling fields. The phase 1 behaviour is similar to the positive exchange bias reported earlier by Yang *et al.*^27^, whereas that of phase 2 is unexpected and different from bifurcated loops reported earlier in systems with macroscopic domains^30^,^31^.

Temperature-dependent hysteresis loops of sample A after field cooling in 15 mT are shown in Fig. 1d (1 μemu=1 nA m^2^). With increasing temperature, the phase 1 exchange bias decreases and vanishes just below the NiCoO Néel temperature of 401 K (Fig. 1e)^26^,^27^, whereas the coercivity remains largely unchanged, as shown in Fig. 1f; the phase 2 exchange bias also decreases, vanishing around 420 K (Fig. 1d). The loop squareness, defined as the ratio of the remanent magnetization *M*_R_ and *M*_S_, measured up to 2 T, exhibits a non-monotonic temperature dependence, as shown in Fig. 1g: first decreasing to 0 at 420 K, coincident with the disappearance of phase 2, then increasing to unity over 450–520 K. This behaviour is consistent with the magnetization of the NiCo being balanced against the GdFe. The *M*_S_ decreases with increasing temperature until 450 K and then increases. For Gd_0.42_Fe_0.58_ we do not expect a compensation point where the Gd and Fe moments cancel out each other^32^. Similar trends are also seen for sample B (*x*=0.48).

**Figure 2:**
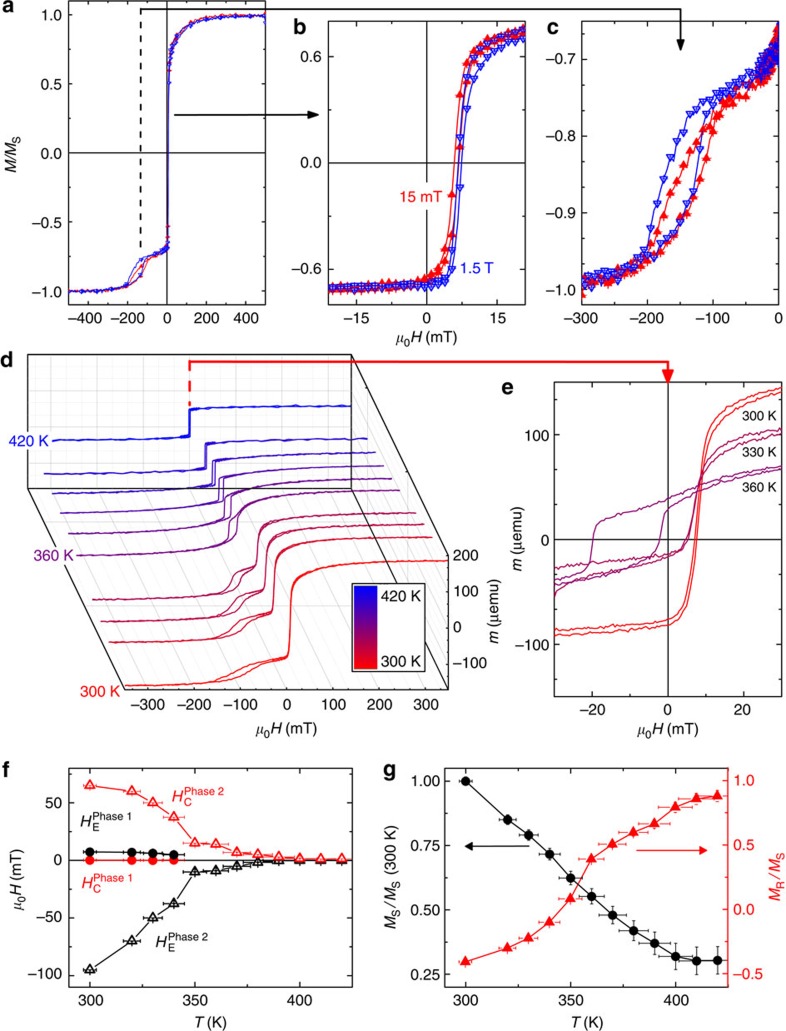
Magnetometry results for sample C (Gd_0.53_Fe_0.47_/NiCoO). (**a**) Room-temperature hysteresis loops under different cooling fields of 15 mT (red) and 1.5 T (blue). (**b**,**c**) Zoomed-in views of phase 1 and phase 2, respectively, as indicated by arrows. Temperature-dependent (**d**) hysteresis loop (300–420 K, marked by the scale bar, with a zoomed-in view shown in **e**), (**f**) coercivity and exchange bias, and (**g**) saturation magnetization and squareness are shown after field cooling in 15 mT. Error bars are determined by the machine sensitivity limits. For clarity, not all data points are shown.

The results for samples C (*x*=0.53) and D (*x*=0.57) are quite different. In these samples, by adjusting the Gd content, the GdFe *T*_C_ is tuned below the NiCoO Néel temperature. Room-temperature hysteresis loops for sample C under different *H*_FC_ are shown in Fig. 2a, which also exhibit two magnetic phases. However, the low anisotropy phase with small coercivity (phase 1, Fig. 2b) is always positively biased, whereas the high anisotropy phase (phase 2, Fig. 2c) exhibits only a single open loop that is always negatively biased. Unlike samples A and B, there is little difference between *μ*_o_*H*_FC_=15 mT and 1.5 T. Temperature-dependent magnetic characteristics for sample C after field cooling in 15 mT are shown in Fig. 2d,e. Phase 1 disappears above 350 K (Fig. 2e), in agreement with *T*_C_ for Gd_0.53_Fe_0.47_ (ref. 32), suggesting that phase 1 can be attributed to the GdFe; in contrast, the exchange bias in phase 2 exhibits a two-step temperature dependence (Fig. 2f): although substantially suppressed beyond the GdFe *T*_C_ of 350 K, it persists until ≈400 K, the NiCoO Néel temperature. Thus, phase 2 is not related to the GdFe but rather to a higher *T*_C_ phase; the two-step dependence suggests that this phase is in contact with both the NiCoO and GdFe, probably at the interface of the two layers. Furthermore, with increasing temperatures the saturation magnetization decreases, while the loop squareness increases (Fig. 2g). Sample D (*x*=0.57) behaves similar to sample C, with the only difference being that the room-temperature coercivity of phase 1 was further reduced. The Gd concentration and cooling field dependence of the magnetic properties are summarized in Supplementary Fig. 1.

### X-ray magnetic circular dichroism

**Figure 3:**
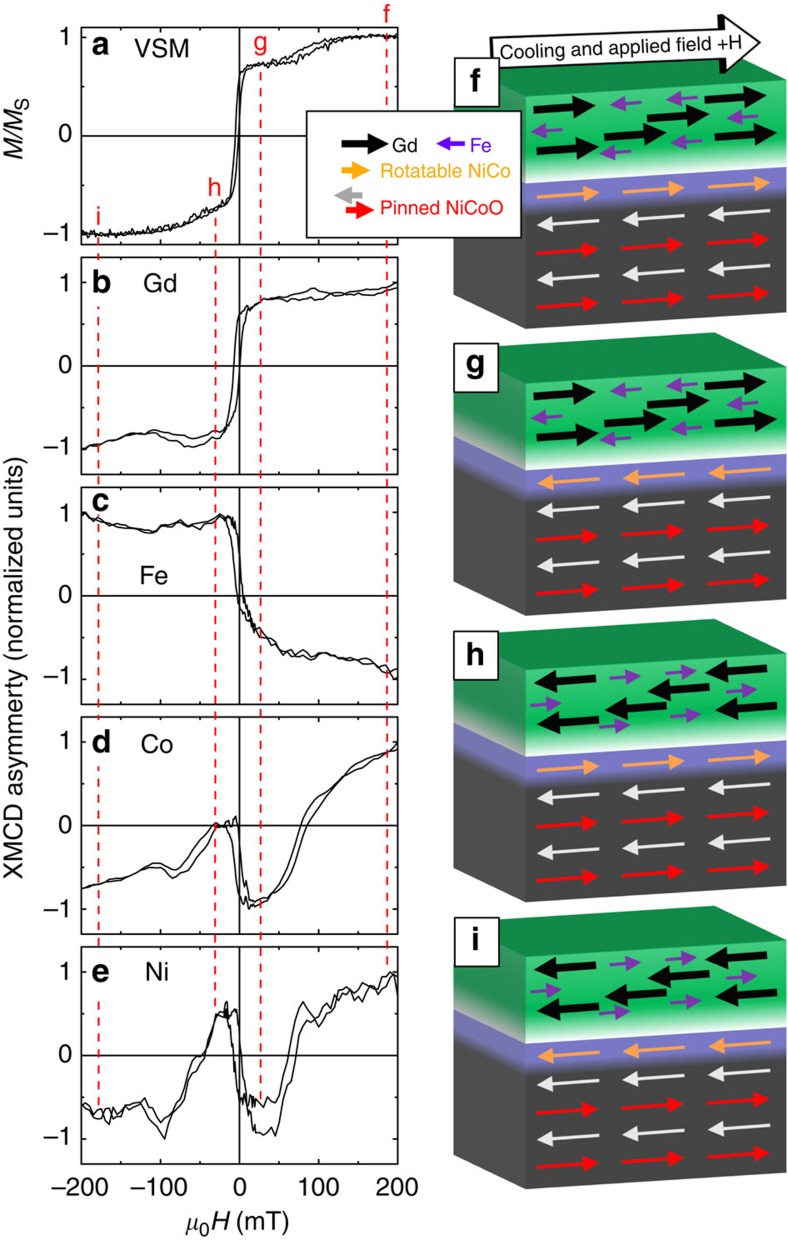
Collective and element-specific magnetic hysteresis loops. Room-temperature hysteresis loops of sample A (*x*=0.42, with a Cu capping layer) measured by (**a**) VSM and element-specific XMCD for (**b**) Gd, (**c**) Fe, (**d**) Co and (**e**) Ni. Schematic illustrations of the sample magnetic configuration at various stages of the reversal are shown in **f**–**i**, respectively. In the layer structure, from top to bottom, the layers are GdFe (green), GdO_*y*_ and GdFe mixture (white), NiCo (blue) and NiCoO (black), respectively. Arrows in the layers indicate the magnetization directions.

To further investigate the origin of phase 2 that gives rise to the subloops at high fields, XMCD was used to extract element-specific hysteresis loops on sample A at room temperature after field cooling in 15 mT, as shown in Fig. 3b–e, along with the vibrating sample magnetometer (VSM) loop in Fig. 3a. For each of the elements probed, the XMCD asymmetry (see Methods) is non-zero, indicating the presence of rotatable ferromagnetic moments (Supplementary Fig. 2). The Gd and Fe loops shown respectively in Fig. 3b,c illustrate the expected ferrimagnet ordering with the Gd moments dominating the Fe ones at room temperature; the correlation with the VSM loop confirms that phase 1 is due to the GdFe. In contrast, the presence of significant uncompensated rotatable Co and Ni moments is rather unexpected. Magnetometry measurements of as-grown NiCoO films (Supplementary Fig. 3) and X-ray photoemission spectroscopy studies of the copper capping layer revealed no appreciable magnetic moments. The XMCD loops for Co and Ni, shown in Fig. 3d,e, respectively, exhibit an interesting dip/bump/dip structure, indicating a complex reversal behaviour where the switching of Co and Ni are at times against the external magnetic field.

From the element-specific hysteresis loops, we can determine the sequence of magnetization reversal from positive saturation, as illustrated in Fig. 3f–i for four representative stages. At high positive fields, the Gd, Ni and Co are all parallel to the applied field, whereas the Fe is opposite, AF coupled to Gd (Fig. 3f). As the field is reduced but remains positive (Fig. 3g), the NiCo reverses, presumably to satisfy the AF exchange coupling to the GdFe, giving rise to the apparent phase 2 subloop. As the applied field becomes negative (Fig. 3h), the Gd reverses and so do the Fe, Ni and Co, such that the Gd remains AF coupled to Fe and NiCo. The reversal of the NiCo against the applied field shows that the NiCo is quite strongly exchange coupled to the GdFe. Finally, at large negative fields (Fig. 3i) the NiCo again becomes parallel to the GdFe, as the field breaks their exchange coupling, leading to the second subloop for phase 2.

These magnetization characteristics are also consistent with the temperature-dependent hysteresis loops shown in Fig. 1. As the temperature increases, below 420 K, the GdFe and NiCo moments are manifested in the two phases, while the GdFe moments dominate; at 420 K, the NiCo and GdFe moments balance out, leading to the appearance of a compensation point (Fig. 1d); above 420 K, NiCo moments become dominant, as the GdFe approaches its *T*_C_.

### Depth profiling with PNR

**Figure 4:**
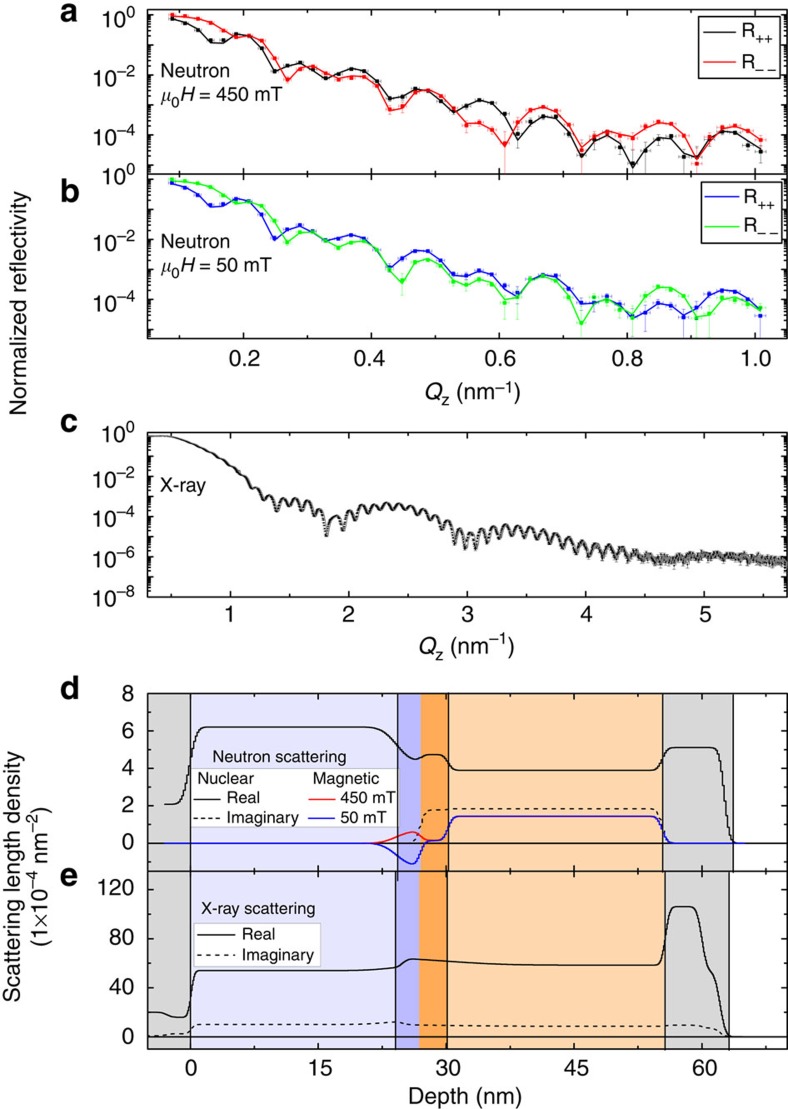
Magnetic and structural depth profiles. PNR data (symbols) and fitted reflectometry (lines) for sample A (*x*=0.42) in a (**a**) 450- and (**b**) 50-mT field applied parallel to the cooling field; (**c**) X-ray reflectometry data (symbols) and fitted reflectometry (line) of sample A. (**d**) The fitted profile determined from PNR with the real (solid) and imaginary (dashed) nuclear structure shown in black and magnetic depth profile in blue (50 mT) and red (450 mT); the structural profile from X-ray reflectivity (XRR) is shown in **e**. For **d** and **e**, the film structure from the left to right, at increasing depth, corresponds to the Si substrate (grey), NiCoO (light blue), interfacial NiCo (dark blue), interfacial GdFe/GdO_*y*_ (peach), GdFe (brown), Ta cap (grey) and air (white). Error bars in *Q*_z_ identify machine precision; error bars in normalized reflectivity are defined by the s.d. and scales with the square root of the number of measurements.

To confirm that the uncompensated elemental NiCo is indeed at the interface, we have employed PNR to probe the nuclear and magnetization depth profiles^33^,^34^,^35^,^36^. The fitted reflectometry data and corresponding profiles for sample A after field cooling in 15 mT, measured at 450 and 50 mT in-plane fields applied parallel to the cooling field, are shown in Fig. 4a,b, respectively. These measurement fields correspond to stages *f* and *g* in Fig. 3, and thus the only difference in the sample magnetic configuration is the reversal of the rotatable NiCo. The plots show a clear, field-dependent difference in the reflectometry for *Q*_Z_>0.4 nm^−1^.

The nuclear depth profile obtained from the neutron reflectivity (Fig. 4d) clearly differentiates the GdFe(O) and NiCo(O) layers by the imaginary component of the nuclear scattering. Moving from GdFe towards the substrate, at the GdFe/NiCoO interface there is a local maximum in the nuclear scattering length density (solid black line). We suggest that the bump can be attributed to GdFeO, where the oxygen increases the nuclear scattering length density relative to its neighbouring GdFe. The imaginary component of the nuclear scattering length density (corresponding to neutron absorbance, generally identifying Gd, as it is a strong neutron absorber) remains constant over the same region (dashed black line), indicating that the Gd remains localized, while it was infiltrated with additional non-absorbent elements (for example, oxygen). Underneath the GdFeO region the imaginary component drops to zero, whereas the real component exhibits a dip, indicating that this region does not contain Gd and can be attributed to elemental NiCo. The magnetic depth profiles measured in 450 and 50 mT (Fig. 4d) show that the magnetic moment drops across the GdFe/NiCoO interface to zero in the NiCoO layer. For the 450 and 50 mT measurements, the interfacial magnetization is parallel and antiparallel to the GdFe layer magnetization, respectively. This difference corresponds to the transition between stages *f* and *g* in Fig. 3, which through XMCD was shown to originate from rotatable NiCo. This last piece of evidence shows that the NiCo responsible for phase 2 (Fig. 1) is indeed located at the interface and is probably the result of oxygen migration from NiCoO to GdFe. For comparison, the X-ray reflectivity spectrum—which is insensitive to magnetic ordering—is shown in Fig. 4c. The structural depth profile determined by X-ray reflectivity in Fig. 4e has features that track those in the PNR depth profile, especially the thicknesses, and shows a slight peak in the scattering length density at the GdFe/NiCoO interface, serving as another confirmation of the structural profile.

Comparing the integrated magnetization change of phase 2 in sample A to the saturation magnetization of the NiCo binary alloy, the interfacial layer thickness can be calculated to be 2.3 nm, consistent with the NiCo thickness obtained from PNR (≈2.5 nm). With increased Gd content, the magnetization associated with phase 2 increases, for example, increasing to 4 nm in sample D. It is worth noting that the FM moments of the interfacial layer are much larger than expected from the interfacial uncompensated AF moment in typical exchange bias systems^37^,^38^.

## Discussion

The mechanisms for the positive and controllable exchange bias can be understood by the AF interfacial exchange coupling between GdFe and uncompensated moments of NiCoO, with the latter existing as pinned and unpinned moments (including the metallic NiCo layer). For samples A and B where the GdFe *T*_C_ is above the NiCoO *T*_N_, the pinned NiCoO moments give rise to the positive exchange bias in GdFe under increasing *H*_FC_ in phase 1. This is the common positive exchange bias due to the AF interfacial exchange coupling, as seen previously^25^,^39^. In the meantime, the unpinned moments, identified as the rotatable NiCo earlier, give rise to the subloops in phase 2, whose reversal are driven by the AF coupling to the Gd and FM coupling to the pinned uncompensated NiCoO moments. Cooling in small *H*_FC_ across the NiCoO *T*_N_, the GdFe moments (dominated by the Gd lattice) are aligned with *H*_FC_; as the NiCoO orders, its uncompensated pinned moments FM couple to the NiCo, which is AF coupled to GdFe, thus ordering opposite to *H*_FC_. This establishes the negative exchange bias in GdFe (phase 1) by the pinned uncompensated NiCoO moments. During the hysteresis loop measurement, the rotatable NiCo moments, forced parallel to the field direction at saturation, switch first to be opposite to the Gd and into alignment with the pinned NiCoO moments, leading to the subloop in the first quadrant (Fig. 1c); as the GdFe reverses, these rotatable NiCo moments switch again in negative fields, leading to the second subloop in the third quadrant. As *H*_FC_ becomes large enough to break the AF coupling, the uncompensated pinned NiCoO moments are forced to be parallel to both the GdFe and *H*_FC_, and this configuration is frozen-in on cooling to room temperature, leading to a positive exchange bias in phase 1. As to the subloops in phase 2, the pinned NiCoO moments now act to stabilize the rotatable NiCo moments, whose reversal under the AF coupling with GdFe occurs at a more negative applied field, leading to the negative shift of the phase 2 loops. Thus, the interfacial NiCo moments and their AF exchange coupling with the GdFe are manifested in the complex exchange bias characteristics.

**Figure 5:**
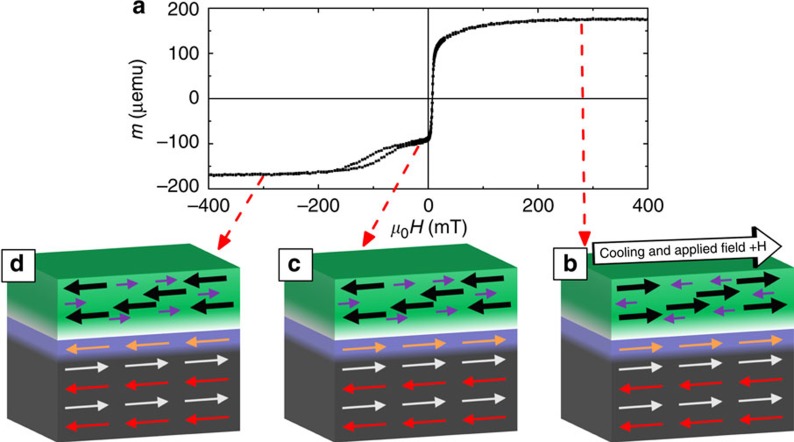
Schematic illustrations of sample C magnetic configurations. Configurations during (**a**) the magnetic hysteresis loop measurement at room temperature are given at (**b**) saturation, (**c**) GdFe reversal and (**d**) NiCo reversal. In the layer structure, from top to bottom, the layers are GdFe (green), GdO_*y*_ and GdFe mixture (white), NiCo (blue) and NiCoO (black), respectively. Arrows in the layers indicate the magnetization directions.

For the case of the higher Gd content samples C and D, the *T*_C_ in GdFe is tuned to be lower than the *T*_N_ of the NiCoO, an unusual situation^40^. Thus, during field cooling approaching the NiCoO *T*_N_, only the rotatable NiCo moments remain FM and their orientation is set exclusively by the cooling field. Consequently, the pinned uncompensated NiCoO moments, through FM coupling to these NiCo, is also aligned with *H*_FC_ and this configuration is frozen-in at room temperature. The GdFe, through its AF coupling with the pinned uncompensated NiCoO moments, is always positively biased, unlike the cases in samples A and B where the positive bias is controllable under different *H*_FC_. An illustration of the different reversal stages during the hysteresis loop measurement (Fig. 5a) at room temperature is shown in Fig. 5. Reversing from positive saturation (Fig. 5b), the GdFe layer is the first to reverse in a positive applied field (Fig. 5c). The rotatable NiCo is stabilized by both the pinned NiCoO moments and the GdFe, until a sufficiently large negative field is applied (Fig. 5d). Thus, phase 2 only exhibits a single subloop, which is always negatively biased.

The interfacial rotatable NiCo layer, with a relatively significant thickness of a few nanometres, plays a prominent role in the complex bias phenomena exhibited in the GdFe/NiCoO system. Control experiments on single-layer films of GdFe measured up to 2 T do not show any two-phase behaviour, consistent with previous results^32^,^41^,^42^, suggesting that the 2 T field is insufficient to break the Gd–Fe AF coupling. Similarly, single-layer films of NiCoO show only a linear magnetic field dependence in their hysteresis loops, characteristic of an AF. These control experiments indicate that the second phase is not intrinsic to each of the as-grown GdFe and NiCoO films, but rather a derived effect of the bilayer construction. It has been previously suggested that Gd is a strong reducing agent for some metal oxides^43^, and that oxygen can be readily moved between GdO_*y*_ and Co^10^. Thus, we suggest that the mechanism at work is a redox reaction between the NiCoO and Gd, forming an interfacial region of elemental NiCo and GdO_*y*_. Furthermore, Gd is also expected to reduce any iron oxide (discussed below), resulting in an interface that is probably a mixture of GdO_*y*_ and residual GdFe (with varying Gd:Fe ratio, even Fe). Athough the GdO_*y*_ locally impedes the interfacial coupling, it is not expected to be continuous enough to completely suppress the exchange bias, which is still mediated through residual GdFe. The oxidized interface thickness and continuity are limited by oxygen diffusion within the GdFe and NiCoO, and thus is likely to be very thin. This scenario is consistent with the PNR profile. The real part of the nuclear scattering length density is shown to increase at the base of the GdFe, presumably due to the incorporation of oxygen, a strong neutron scatterer, whereas the imaginary part (which depends almost entirely on the Gd volume concentration) remains constant. At the same time, the nuclear scattering length density below the GdFeO is lower than its neighbouring NiCoO due to the loss of its oxygen, consistent with the required balancing of the redox equation.

To further investigate the plausibility of this explanation, the net heat of formation and Gibbs free energy at room temperature were calculated for 3NiO (CoO)+2Gd→Gd_2_O_3_+3Ni (Co) and were found to be around −1.1 MJ mol^−1^ for both terms^44^, indicating that the reaction will occur spontaneously. Similar calculations for the formation of iron oxide (CoO+Fe→FeO_*x*_+Co) yield Gibbs free energies in the range of −30 to −160 kJ mol^−1^ for FeO, Fe_2_O_3_ and Fe_3_O_4_ (ref. 44), again indicating a spontaneous reaction. However, iron oxide is strongly reduced by Gd (FeO_*x*_+Gd→Gd_2_O_3_+Fe, Gibbs free energy of about −1.0 MJ mol^−1^). Thus, the calculated results support the spontaneous oxidation of Gd at the interface and reduction of the NiCoO. This type of interfacial redox behaviour has been seen previously in NiO/Co bilayer films^45^, for which similar calculations also support spontaneous oxygen migration to the interfacial Co.

For samples A and B, the bias of the phase 2 subloops reflect the AF exchange interaction between the NiCo and GdFe, as well as the FM exchange interaction between the NiCo and the NiCoO. As the orientation of the pinned uncompensated NiCoO moments does not change with applied field after field cooling to room temperature, and the GdFe orientation does, the bias from the GdFe (*H*_E_^*NiCo/GdFe*^) changes on field cycling and the bias from the NiCoO (*H*_E_^*NiCo/NiCoO*^) remains constant. Thus, the bias field for the *+H* subloop is determined to be *H*_E_^*+H*^=*H*_E_^*NiCo/NiCoO*^+*H*_E_^*NiCo/GdFe*^, whereas the *−H* subloop is biased by *H*_E_^*−H*^=*H*_E_^*NiCo/NiCoO*^−*H*_E_^*NiCo/GdFe*^. Therefore, the bias fields can be separated: *H*_E_^*NiCo/NiCoO*^=(*H*_E_^*+H*^+*H*_E_^*−H*^)/2 and *H*_E_^*NiCo/GdFe*^=(*H*_E_^*+H*^−*H*_E_^*−H*^)/2. These values are determined to be 56 and −169 mT for sample A, and 36 and −184 mT for sample B, respectively, after field cooling in 1.5 T. Using the generalized Meiklejohn–Bean approach^46^, the exchange energy density is calculated to be |*J*^*NiCo/NiCoO*^ *S*_*NiCo*_ *S*_*NiCoO*_|=1.3 × 10^*−*4^ J m^−2^ and |*J*^*NiCo/GdFe*^ *S*_*NiCo*_ *S*_*GdFe*_|=-3.8 × 10^*−*4^ J m^*−*2^ for sample A, and 1.1 × 10^*−*4^ and −5.7 × 10^*−*4^ J m^*−*2^ for sample B, respectively. These values are much smaller than the bulk exchange values (scaled by the interface number density and atomic spin moment) for Co–Co (7 × 10^*−*3^ J m^*−*2^) and Gd–Co (−2 × 10^*−*3^ J m^*−*2^) (ref. 32), probably due to the interface details, as is typical in exchange-biased systems.

For samples C and D, the second phase is always negatively biased (there is no *H*_E_^*+H*^ feature or any *H*_FC_ dependence); thus, the contributions of NiCoO and GdFe exchange coupling cannot be separated as above. However, the loss of the two subloop features does imply that the NiCo remains robust against the AF exchange coupling with the GdFe, indicating that *H*_E_^*NiCo/GdFe*^<*H*_E_^*NiCo/NiCoO*^. As *H*_E_^*NiCo/NiCoO*^ is not expected to change with GdFe stoichiometry, we can conclude that the NiCo/GdFe exchange coupling decreases with increased Gd in GdFe. The net exchange fields were extracted from the major loops to be 143 and 78 mT for samples C and D, respectively, under 1.5 T cooling field. As there is only one subloop in samples C and D, *H*_E_^*NiCo/NiCoO*^ and *H*_E_^*NiCo/GdFe*^ cannot be separated; the net coupling energy density is 5.4 × 10^*−*4^ and 3.5 × 10^*−*4^ J m^*−*2^, respectively.

In summary, we have demonstrated effective magneto-ionic manipulation of the Gd_*x*_Fe_1−*x*_/NiCoO interfaces and directly observed oxygen migration across buried interfaces and the impacts on the controlled positive exchange bias. The complex magnetization reversal is manifested in the hysteresis loops as multiple phases in bilayer samples with 42 and 48 at.% Gd: phase 1, identified as a single low anisotropy loop, was shown by XMCD spectroscopy to originate primarily from reversal of the GdFe, whereas phase 2, consisting of a pair of asymmetrically biased subloops, was shown to originate from reversal of rotatable NiCo moments. By varying the cooling field, the bias of phase 1 and asymmetry of phase 2 were shown to shift, with opposite trends. This controllability was suppressed and eventually destroyed by increasing the Gd content to 53 and 57 at. %, which lowered the GdFe Curie temperature below the NiCoO Néel temperature. The AF exchange coupling between the interfacial NiCo and GdFe causes the NiCo moments to be parallel to the GdFe at high fields and to be antiparallel at low fields. The field-dependent orientation of the interfacial moments controls the AF orientation and the corresponding bias field direction. The interfacial NiCo was attributed to a redox reaction between the NiCoO and GdFe, leading to the formation of NiCo and GdO_*y*_. These results demonstrate an effective way to tailor the interfacial characteristics and interlayer exchange coupling in metal/oxide heterostructures. Reversible control of the oxygen migration in such systems, for example, using an electric field, may enable concepts for energy-efficient spintronic devices.

## Methods

### Sample fabrication

Bilayer films of Ni_0.47_Co_0.53_O (20 nm)/Gd_*x*_Fe_1−*x*_ (30 nm) (*x*=0.42–0.57) were magnetron-sputtered on naturally oxidized Si (100) wafers at ambient temperature in 0.33 Pa Ar in a high-vacuum chamber (base pressure <6.7 × 10^*−*6^ Pa). The NiCoO layer was radio frequency sputtered from a pressed composite target of CoO and NiO powders, while the GdFe was direct current co-sputtered from elemental targets. The samples were capped with 6 nm of Ta (or Cu for the XMCD samples).

### Characterizations

X-ray diffraction revealed polycrystalline NiCoO and amorphous/nanocrystalline GdFe. Stoichiometry of the NiCoO was determined by energy dispersive X-ray spectroscopy, along with analysis of the nuclear scattering length density and bulk number density, to be Ni_0.47_Co_0.53_O and the GdFe to be Gd_0.42_Fe_0.58_, Gd_0.48_Fe_0.52_, Gd_0.53_Fe_0.47_ and Gd_0.57_Fe_0.43_ (identified as samples A–D, respectively). Magnetic measurements were performed using a VSM with the field parallel to the cooling field axis, unless otherwise noted. Element-specific hysteresis loops were measured by XMCD at the Advanced Light Source Beamline 6.3.1. Loops for Fe, Ni, Co and Cu were determined by tuning to their respective *L*_2,3_ edges, whereas Gd loops were determined by tuning to the *M*_4,5_ edge, following previously outlined procedures^29^,^37^,^47^. Magnetic contrast was achieved by measuring the fluorescence yield signal with the left and right circularly polarized X-rays at 30° grazing incidence. XMCD asymmetry is achieved by calculating the difference of the left and right circularly polarized signals.

Polarized neutron reflectivity was used to probe depth-dependent nuclear and magnetic profiles of the films, performed on the polarized beam reflectometer and the multi-angle grazing-incidence k-vector reflectometer at the NIST Center for Neutron Research using wavelength *λ*=0.475 nm neutrons. The applied magnetic field and corresponding neutron spin direction are in-plane and parallel to the field cooling direction. The reflectometry data are presented for the non-spin flip cases, with incident and scattered neutrons having the same spin, identified for the case of spin-up (down) by *R*_*++*_ (*R*_*−−*_). This configuration is sensitive to in-plane magnetization along the neutron spin direction. The spin flip reflectometry, which is sensitive to a net in-plane magnetization orthogonal to the applied field, showed no appreciable signal. Profile fitting was performed using the Refl1D software package^48^. Each fitted model consisted of the GdFe, NiCoO and Ta capping layers, as well as interfacial NiCo and GdO_*y*_ layers; all the measurements were fitted simultaneously, with the structural parameters between different models constrained to be the same. Alternative PNR fitting were also carried out for comparison, without the interfacial layer, and the resultant fits were significantly worse (Supplementary Fig. 4 and Supplementary Note 1). X-ray and neutron reflectometry data are presented with respect to the momentum transfer vector, *Q*.

## Additional information

**How to cite this article:** Gilbert, D. A. *et al.* Controllable positive exchange bias via redox-driven oxygen migration. *Nat. Commun.* 7:11050 doi: 10.1038/ncomms11050 (2016).

## Supplementary Material

Supplementary Information Supplementary Figures 1-4 and Supplementary Note 1.
