# Supplementary material for: Controllable positive exchange bias via redox-driven oxygen migration
Source: Nat Commun. 2016 Mar 21;7:11050. doi: 10.1038/ncomms11050 (PMC4802176; doi:10.1038/ncomms11050)
Supplement: Supplementary Information — Supplementary Figures 1-4 and Supplementary Note 1. [file ncomms11050-s1.pdf]

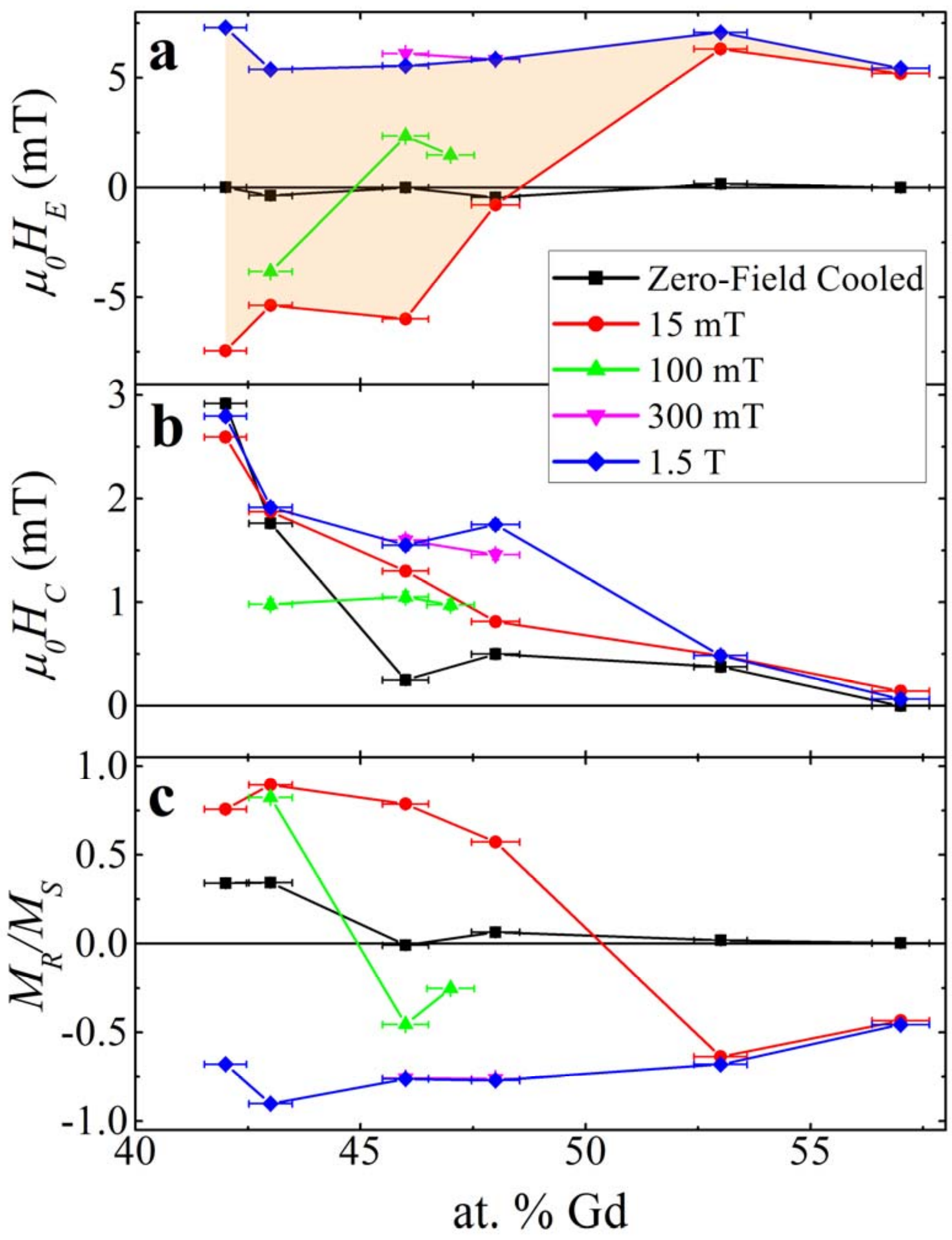

Supplementary Figure 1. Magnetic property dependence of Gd_xFe_{1-x} /NiCoO on the Gd concentration and H_{FC}. Results are shown for (**a**) the phase 1 bias, (**b**) major loop coercivity and (**c**) remanence. Controllability is realized in samples with at. Gd < 53%.

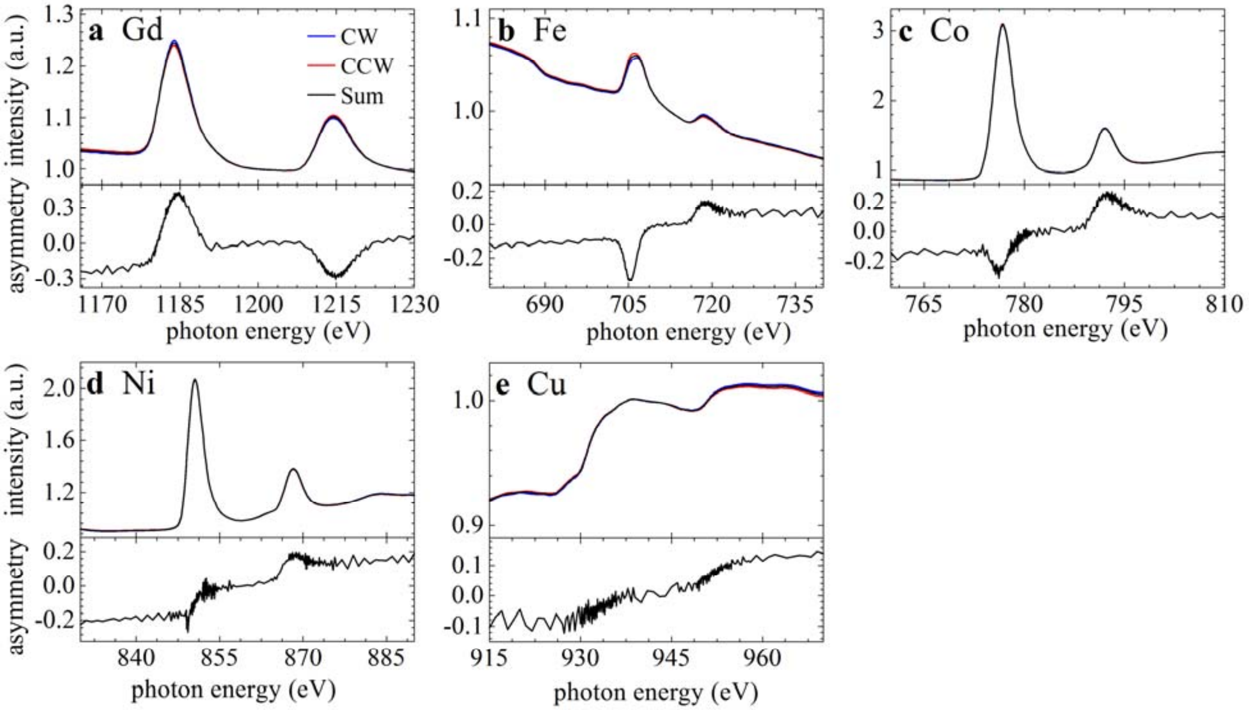

Supplementary Figure 2. **X-ray Absorption Spectroscopy and XMCD asymmetry for sample A**. Results are shown for (**a**) Gd, (**b**) Fe, (**c**) Co, (**d**) Ni, and (**e**) Cu. The X-ray absorption spectroscopy pattern (top panel) shows signal from each element; XMCD signal (bottom panel) shows a magnetic response from the Gd, Fe, Co, and Ni, and no response from the Cu.

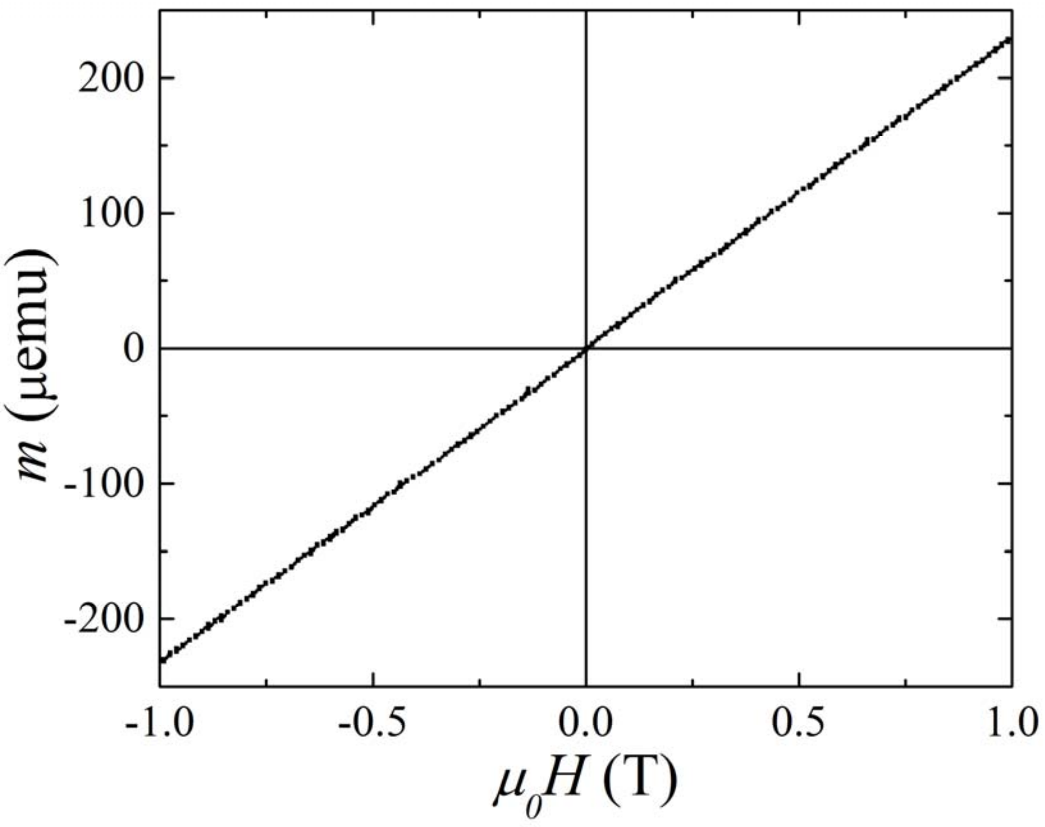

Supplementary Figure 3. **Magnetometry of a single thin film of NiCoO (30nm)/Si.** The response shows a typical antiferromagnet behavior.

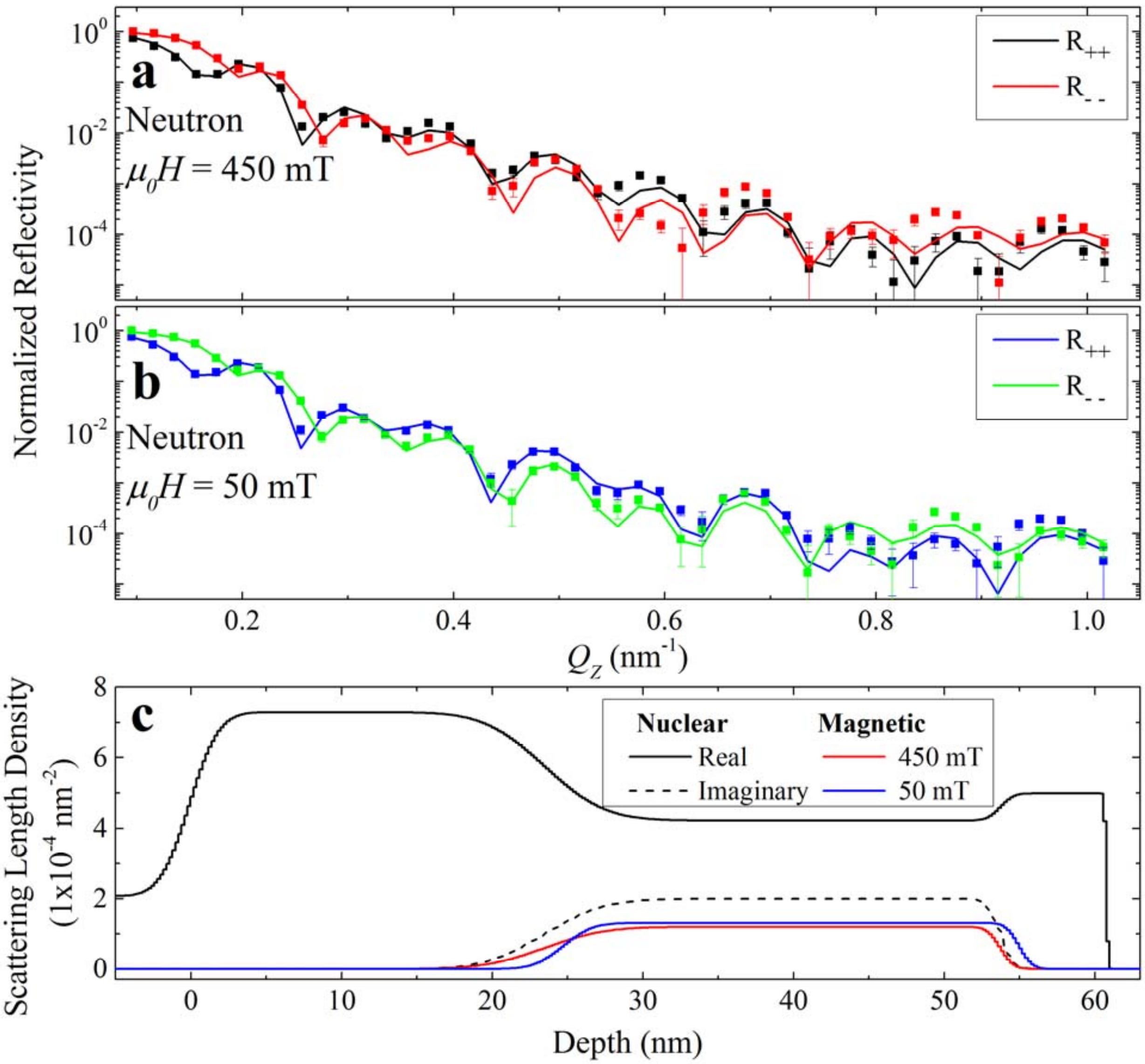

Supplementary Figure 4. **Alternative PNR Model.** Polarized neutron reflectometry data (symbols) and fitted reflectometry (lines) for sample A (x=0.42) in a (**a**) 450 mT and (**b**) 50 mT field applied parallel to the cooling field; (**c**) The fitted profile determined from PNR with the real (solid) and imaginary (dashed) nuclear structure shown in black and magnetic depth profile in blue (50 mT) and red (450 mT). The model in these fits does not include an interfacial layer.

Supplementary Note 1

Alternative models were considered for the neutron reflectometry data, in support of the uniqueness of the presented model. These alternative models were significantly worse at matching the data. One such model presented in Supplementary Figure 4 is the nominal bilayer structure of NiCoO/GdFe - without the interfacial layer. Clearly the fit is much worse than that shown in the main text. This is quantitatively supported in that the χ^2 of the model in the main text is 3.8, while the one in the figure above is 35.
